# Beta-band power modulation in the human amygdala differentiates between go/no-go responses in an arm-reaching task

**DOI:** 10.1088/1741-2552/ad5ebe

**Published:** 2024-07-16

**Authors:** Ryan S Chung, Roberto Martin del Campo Vera, Shivani Sundaram, Jonathon Cavaleri, Zachary D Gilbert, Andrea Leonor, Xiecheng Shao, Selena Zhang, Alexandra Kammen, Xenos Mason, Christi Heck, Charles Y Liu, Spencer S Kellis, Brian Lee

**Affiliations:** 1 Department of Neurological Surgery, Keck School of Medicine of USC, University of Southern California, Los Angeles, CA, United States of America; 2 USC Neurorestoration Center, Keck School of Medicine of USC, Los Angeles, CA, United States of America; 3 Department of Neurology, Keck School of Medicine of USC, University of Southern California, Los Angeles, CA, United States of America; 4 Keck School of Medicine of USC, University of Southern California, Los Angeles, CA, United States of America; 5 Viterbi School of Engineering, University of Southern California, Los Angeles, CA, United States of America

**Keywords:** beta-band power, stereotactic electroencephalography (SEEG), neural modulation, amygdala, arm-reaching movements (ARMs), center-out task

## Abstract

*Objective*. Traditionally known for its involvement in emotional processing, the amygdala’s involvement in motor control remains relatively unexplored, with sparse investigations into the neural mechanisms governing amygdaloid motor movement and inhibition. This study aimed to characterize the amygdaloid beta-band (13–30 Hz) power between ‘Go’ and ‘No-go’ trials of an arm-reaching task. *Approach*. Ten participants with drug-resistant epilepsy implanted with stereoelectroencephalographic (SEEG) electrodes in the amygdala were enrolled in this study. SEEG data was recorded throughout discrete phases of a direct reach Go/No-go task, during which participants reached a touchscreen monitor or withheld movement based on a colored cue. Multitaper power analysis along with Wilcoxon signed-rank and Yates-corrected *Z* tests were used to assess significant modulations of beta power between the Response and fixation (baseline) phases in the ‘Go’ and ‘No-go’ conditions. *Main results*. In the ‘Go’ condition, nine out of the ten participants showed a significant decrease in relative beta-band power during the Response phase (*p* ⩽ 0.0499). In the ‘No-go’ condition, eight out of the ten participants presented a statistically significant increase in relative beta-band power during the response phase (*p* ⩽ 0.0494). Four out of the eight participants with electrodes in the contralateral hemisphere and seven out of the eight participants with electrodes in the ipsilateral hemisphere presented significant modulation in beta-band power in both the ‘Go’ and ‘No-go’ conditions. At the group level, no significant differences were found between the contralateral and ipsilateral sides or between genders. *Significance.* This study reports beta-band power modulation in the human amygdala during voluntary movement in the setting of motor execution and inhibition. This finding supplements prior research in various brain regions associating beta-band power with motor control. The distinct beta-power modulation observed between these response conditions suggests involvement of amygdaloid oscillations in differentiating between motor inhibition and execution.

## 1. Introduction

The human amygdala consists of a set of medial temporal lobe nuclei and is best known for its roles in processing reward-based behaviors and fearful stimuli [1–3]. Though many studies have explored the involvement of cortical structures in motor planning and execution [4–7], there has been little investigation into the contribution of subcortical structures, such as the amygdala, to these motor processes. Emerging evidence has shown connectivity between the amygdala and motor areas, suggesting that the amygdala may play a role in modulating human motor function. For example, a diffusion-weighted magnetic resonance imaging (MRI) and probabilistic tractography study in humans showed structural connectivity between the amygdala and motor cortex via the external capsule tracts [8]. In addition, Toschi *et al* uncovered a functional connection between the amygdala and the premotor circuit in humans [9]. Functional MRI (fMRI) studies in humans have also demonstrated functional connectivity between the amygdala and various subcortical structures known to play roles in motor processing including the supplementary motor area (SMA) [10], subthalamic nucleus (STN) [10, 11], and globus pallidus [12]. The amygdala has also been implicated in pathological conditions involving these structures. One study utilized resting-state fMRI analysis to show increased connectivity between the amygdala and putamen in Parkinson’s disease (PD) patients with freezing of gait symptoms, further suggesting a potential role of amygdaloid-striatal circuits in motor control [13]. However, the exact role of the amygdala in movement planning, inhibition, and execution remains unclear.

Noninvasive cranial monitoring methods, such as fMRI, have been utilized to visualize cortical activity during motor and sensory functions, providing valuable information on the neural underpinnings of these processes [14]. In comparison with noninvasive methods, direct intracranial neural recordings, such as with stereotactic electroencephalography (SEEG), support significantly greater temporal resolutions and signal-to-noise ratios [15, 16]. Furthermore, SEEG provides access to intracranial recordings with lower hemorrhagic and infectious complication rates than other invasive monitoring methods such as subdural electrocorticography grids [17]. These advancements in electrode technology and signal analysis techniques have furthered our understanding of human behavior, as they allow us to analyze neural electrical signals during both cognitive and motor tasks [18, 19].

Several studies have utilized electrophysiology to study cortical beta-band power (13–30 Hz) modulations in cortical motor processing. Specifically, these studies have shown that beta-band power decreases are associated with movement execution [20, 21] while beta-band power increases are related to movement inhibition [20]. Our group expanded upon these previous studies by using SEEG to investigate beta-band power modulation in the hippocampus, a subcortical structure, in patients with epilepsy during a direct reaching task [22, 23]. In these studies, our group showed that hippocampal beta-band power significantly decreases during the execution of arm-reaching movements (ARMs). Together, these studies suggest that the beta-band is associated with movement inhibition.

Although much of the work characterizing beta-band modulation in motor processing has focused on human and primate cortices [20, 24], recent studies have begun to focus on investigating beta-band activity in subcortical structures [25, 26]. In humans, Kühn *et al* recorded local field potentials (LFP) from the STN of Parkinson’s disease patients performing a Go/No-go task, which required participants to choose between movement inhibition or execution based on a specific cue [21]. For the ‘Go’ condition, they observed decreases in beta-band power in the STN during movement execution and late rebounds in beta-band power after movement concluded [21]. For the ‘No-go’ condition, they observed increases in beta-band power with movement inhibition [21]. Similarly, our group used a Go/No-go task to study how human hippocampal beta-band power changes during movement [27]. In our study, we utilized SEEG recordings from ten participants with drug-resistant epilepsy. We found that eight out of ten participants showed significant decreases in beta-band power during the ‘Go’ movement execution phase [27]. Furthermore, eight out of ten participants showed significant beta-band power increases in the ‘No-go’ movement inhibition phase [27]. These findings are in line with Kühn *et al*, suggesting that the beta-band can signal a movement-related state and modulate according to inhibitory inputs. Yet, to the best of our knowledge, there has not been a study characterizing beta-band power changes with motor execution and inhibition in the human amygdala, despite there being evidence of functional connectivity between the amygdala and motor structures [28] as well as the STN [11].

The laterality of neural oscillations associated with motor processing has been a growing area of investigation. In 2000, Haaland *et al* found that stroke patients with left hemispheric damage had bilateral motor deficits, while those with right hemisphere damage had contralateral motor deficits [29], suggesting an asymmetric interpretation of motor control in the brain. In 2005, Bai *et al* conducted an analysis of beta-band oscillations with surface EEG data from right-handed subjects during a finger-movement task [30]. They found that, in the period just prior to movement, there was significant contralateral event-related desynchronization (ERD) in the beta frequency range during right-hand finger movements [30]. In contrast, during left-hand finger movements, there was significant bilateral ERD in the beta frequency range [30]. To our knowledge, there have been no studies studying the laterality of movement with SEEG recordings, especially within subcortical structures like the amygdala.

Gender-specific differences in neural oscillations have also been observed in several studies. Taylor *et al* studied the dynamics of neural oscillation and observed sex-specific differences in the alpha-, beta-, and theta-bands during adolescent development [31]. In addition, our group previously discovered that female participants showed increased beta-band power modulation in the hippocampus compared to males during a Go/No-go task [27]. Specifically in the gamma-band, sex-specific differences have also been witnessed in visual tasks [32], auditory tasks [32, 33], and in pathological conditions like schizophrenia [34, 35]. However, to our knowledge, there has been no direct investigation into gender-specific differences of gamma-band power in the amygdala during movement.

Our group previously utilized SEEG recordings to show gamma-band modulation in the amygdala during volitional movements associated with a center-out direct reach task, findings that are consistent with the proposed pro-kinetic role of gamma band oscillations in the cortex [36]. Thus, in this study, we aimed to characterize how beta-band oscillations are associated with motor inhibition and execution within the human amygdala. We additionally aimed to analyze differences in laterality of movement processing within the amygdala, as well as in male versus female groups. We chose to focus on the beta-band given its associations with motor processing [20, 21, 37, 38], and leveraged SEEG for its millisecond-scale temporal resolution [14, 39]. In addition, we selected the Go/No-go behavioral paradigm as it is a well-established method to assess motor response inhibition [27, 40]. We hypothesize that, in the amygdala, beta-band power will increase in the setting of movement inhibition and decrease in the setting of movement execution. Furthermore, in line with previous studies, we hypothesize the presence of a lateralization and gender-specific effect of amygdaloid beta-band power in the setting of movement processing.

## 2. Methods

### 2.1. Participants

Ten participants (four female, six male) diagnosed with drug-resistant epilepsy implanted with stereotactic depth (intracranial) electrodes as part of their medical workup for seizure localization were enrolled in this study. The age among all participants ranged from 21 to 46 years (mean 32.6 years) at the time of the study. Individual participant profiles are detailed in table 1. The number and placement location of electrodes were personalized to each participant in accordance with standard of care clinical criteria based upon MRI, positron emission topography (PET) scans, video-EEG monitoring, and seizure semiology by the University of Southern California (USC) Comprehensive Epilepsy Center neurologists, epileptologists, neuroradiologists, and neurosurgeons.

**Table 1.** Individual information for the ten participants with drug-resistant epilepsy who underwent stereotactic depth (intracranial) electrode implantation as part of their medical workup for seizure localization.

Participant profiles					
ID	Gender	Age	Handedness	Pathology	Seizure-onset zone
1	F	45	Right	Not available	Right insula and frontal operculum
2	F	46	Right	Not available	Right orbitofrontal
3	F	21	Right	Not available	Not localized
4	F	21	Right	Right Parahippocampal Gyrus Cavernous Malformation	Right Orbitofrontal
5	M	22	Left	Right Hippocampal Mesial Sclerosis	Not available
6	M	35	Right	Not available	Right Anterior Hippocampus
7	M	31	Right	Not available	Right Amygdala
8	M	39	Right	Not available	Right Orbitofrontal and Right Anterior Hippocampus
9	M	33	Right	Not available	Left Mesial Temporal, Left Hippocampus, and Left Amygdala
10	M	33	Right	Not available	Not available

Electrode placement and contact number varied among participants. Six participants had depth electrodes implanted bilaterally in the amygdala. Two participants had a depth electrode implanted in the left amygdala and another two participants had a depth electrode implanted in the right amygdala. A detailed description of the electrode placement for each participant, including their cerebral laterality and the type and number of electrode leads implanted, is summarized in table 2. Appropriate consent was obtained as approved by the Institutional Review Board (IRB) of the University of Southern California (USC) Health Science Campus (Study ID: HS-17-00554).

**Table 2.** Summary of total electrodes implanted, organized by cerebral laterality (left/right amygdala). The final column lists the number of contacts confirmed to reside within the amygdala using merged pre-operative MRI and post-operative CT imaging.

Implanted electrodes				
ID	Cerebral hemisphere	Number of electrode leads in the amygdala	Type of electrode lead in the amygdala	Number of contacts in the amygdala
1	Left	1	MM16 A-SP05X-000	4
	Right	1	MM16 A-SP05X-000	4

2	Left	1	MM16 A-SP05X-000	4
	Right	1	MM16 A-SP05X-000	4

3	Left	1	MM16 A-SP05X-000	4
	Right	1	MM16 A-SP05X-000	3

4	Left	1	MM16 A-SP05X-000	4
	Right	1	MM16 A-SP05X-000	3

5	Left	1	SD10R-SP05X-000	3
	Right	1	SD10R-SP05X-000	3

6	Left	0	N/A	N/A
	Right	1	SD10R-SP05X-000	4

7	Left	0	N/A	N/A
	Right	1	SD10R-SP05X-000	4

8	Left	1	MM16 A-SP05X-000	4
	Right	0	N/A	N/A

9	Left	1	SD06R-SP05X-000	4
	Right	0	N/A	N/A

10	Left	1	SD10R-SP05X-000	1
	Right	1	SD10R-SP05X-000	3

ALL	Left (8 participants)	8 leads		28 Left Amygdaloid contacts
	Right (8 participants)	8 leads		28 Right Amygdaloid contacts

### 2.2. Signal acquisition and processing

In five participants, the depth electrodes used for recording LFPs in amygdala structures consisted of Macro–Micro depth electrodes. In the remaining participants, Spencer probe depth 1 × 6 or 1 × 10 electrodes were used for recording. The Macro–Micro depth electrodes had six macro and ten micro platinum contacts (MM16 A-SP05X-000, Ad-Tech Medical Instrumentation Corporation, Oak Creek, WI, USA). The macro contacts were 1.57 mm in length and 1.3 mm in diameter, with 5 mm spacing (center-to-center). The Spencer probe depth 1 × 10 electrodes had 10 platinum contacts that were 2.41 mm in length, 1.12 mm in diameter, with 5 mm spacing center-to-center, (SD10R-SP05X-000, Ad-Tech Medical Instrumentation Corporation, Oak Creek, WI, USA). The Spencer probe depth 1 × 6 electrode had the same specifications as the Spencer probe depth 1 × 10 but with only 6 contacts (SD06R-SP05X-000, Ad-Tech Medical Instrumentation Corporation, Oak Creek, WI, USA).

The extracellular potentials recorded with the Spencer depth electrodes and the macro contacts of the Macro-Micro depth electrodes were used for analysis. All the neural signals were amplified with unity gain, filtered with Butterworth 1st order analog high-pass (0.3 Hz) and 3rd order analog low-pass (7500 Hz) filters, digitized with 16-bit, 250 nV resolution, and sampled at 30 000 samples per second using a Neural Signal Processor (NSP; NeuroPort System, Blackrock Microsystems, Salt Lake City, UT, USA). The same signal was anti-aliased with a 500-Hz lowpass filter and downsampled to 2000 samples per second by the NSP. Built-in adaptive line (60 Hz) noise cancellation filtering (4th order hi/lo pass digital filtering) was used for all channels and the online reference contact was an electrode contact located in white matter. The analysis of the present study was performed on the 2000 samples per second recording.

### 2.3. Experimental task design

**Figure 1.**
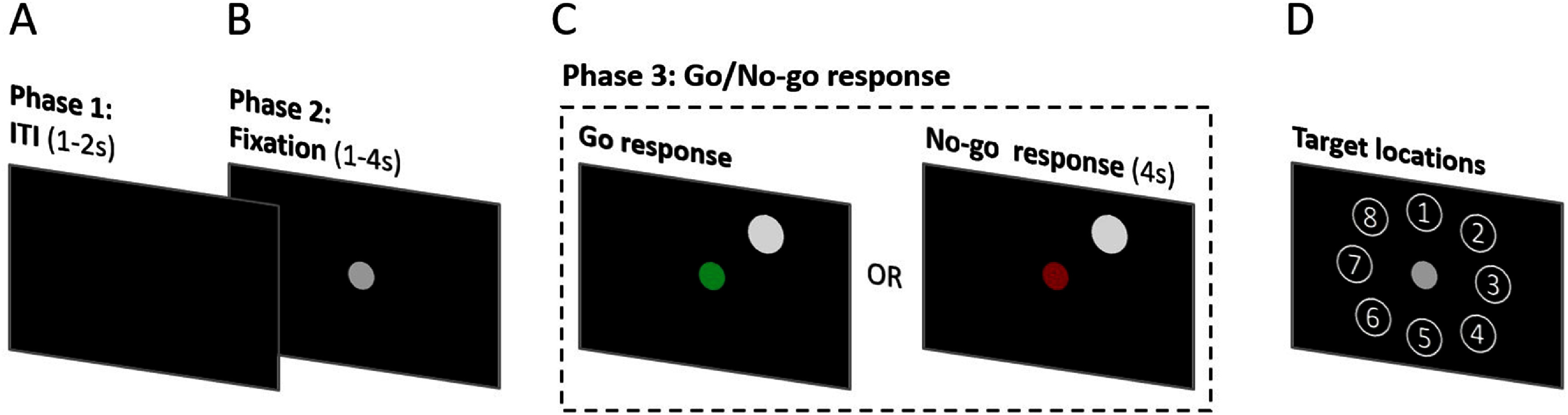
Experimental design of the center-out direct reach Go/No-go task. (A) During the inter-trial interval (ITI) phase the participants prep their hands on the center of the screen and wait for fixation dot (1–2 s). (B) During the fixation phase, the participants point to a fixation dot and fix their gaze on it (1–4 s). ((C). Left panel) participants are cued with a green dot to reach and double tap to a target during the ‘Go’ response (32 trials). ((C). Right panel) during the ‘No-go’ response, participants are cued with a red dot to withhold movement (32 trials). (D) Targets were presented pseudo randomly and balanced in 8 different positions equidistantly from the fixation dot (8 trials per target location).

The participants were asked to perform the Go/No-go center-out direct reach experimental task used in our previous study [27], which consisted of three distinct phases illustrated in figure 1. The phase lengths of the task varied to prevent participants from timed responses. The task was programmed in MATLAB^©^ (2022b, The MathWorks, Inc., Natick, Massachusetts, USA) along with the Psychophysics Toolbox Version 3 (PTB-3). The movement task was presented on a 21.5-inch LED-backlit touch-screen monitor with 1920 × 1080 pixels and 250 cd m^−2^ luminance (S2240Tb, Dell Inc., Round Rock, TX, USA).

The task began with the inter-trial interval (ITI) phase (figure 1(A)), in which no visual cue was presented on the screen for 1–2 s. During this phase, participants had their right hand about 2 inches away from the center of the screen. Next, in the Fixation phase (figure 1(B)), a gray fixation dot of 9.53 mm radius was displayed at the center of a touch screen. Participants were asked to point to the fixation dot with their fingertips, maintain hand position 2 inches from the screen, and fix their gaze on the dot until cued otherwise (1–4 s). Because participants were asked not to move their arms, the fixation phase served as a baseline period to measure modulations of beta-band power spectrum. Finally, in the response phase (figure 1(C)), a white target circle (radius 15.88 mm) pseudo-randomly appeared at one of eight locations equidistantly spaced around the fixation dot (figure 1(D)), with 114.3 mm of center-to-center spacing from fixation dot to target. Simultaneous with the target display, the fixation dot changed to either green (‘Go’ condition) or red (‘No-go’ condition). In the ‘Go’ condition (left column of figure 1(C)), participants moved their right arm towards the target on the screen and double tapped on it. The response time for the ‘Go’ condition is the time difference from the target appearance to the participant’s double tap. In the ‘No-go’ condition (right column of figure 1(C)), the participants withheld their movement towards the target, remaining in the same arm position as the Fixation phase. The response time for the ‘No-go’ condition was fixed to 4 sec beginning at target appearance. The targets were shown in pseudorandom order, with 8 trials per each of 8 target locations (*N* = 64 total trials). Half of the trials (*N* = 32) included a ‘Go’ response and half included a ‘No-go’ response.

### 2.4. Criteria and considerations for data analysis

Trial success was defined as either having a correct double tap on the target in the ‘Go’ condition or a correct absence of motion in the ‘No-go’ condition. Participants were monitored by researchers during the task, recorded by video, and wore a three-axis accelerometer to ensure that there were no aberrant movements not required by the task condition. Trials in which participants moved during the fixation phase or during the ‘No-go’ response were removed from analysis. Additionally, trials were removed if participants failed to double tap on the target during the ‘Go’ condition of the response phase, had a response time of less than 200 ms (deemed accidental), or presented neural data spectral power values exceeding 1.5 times the interquartile range beyond the first and third quartiles.

**Figure 2.**
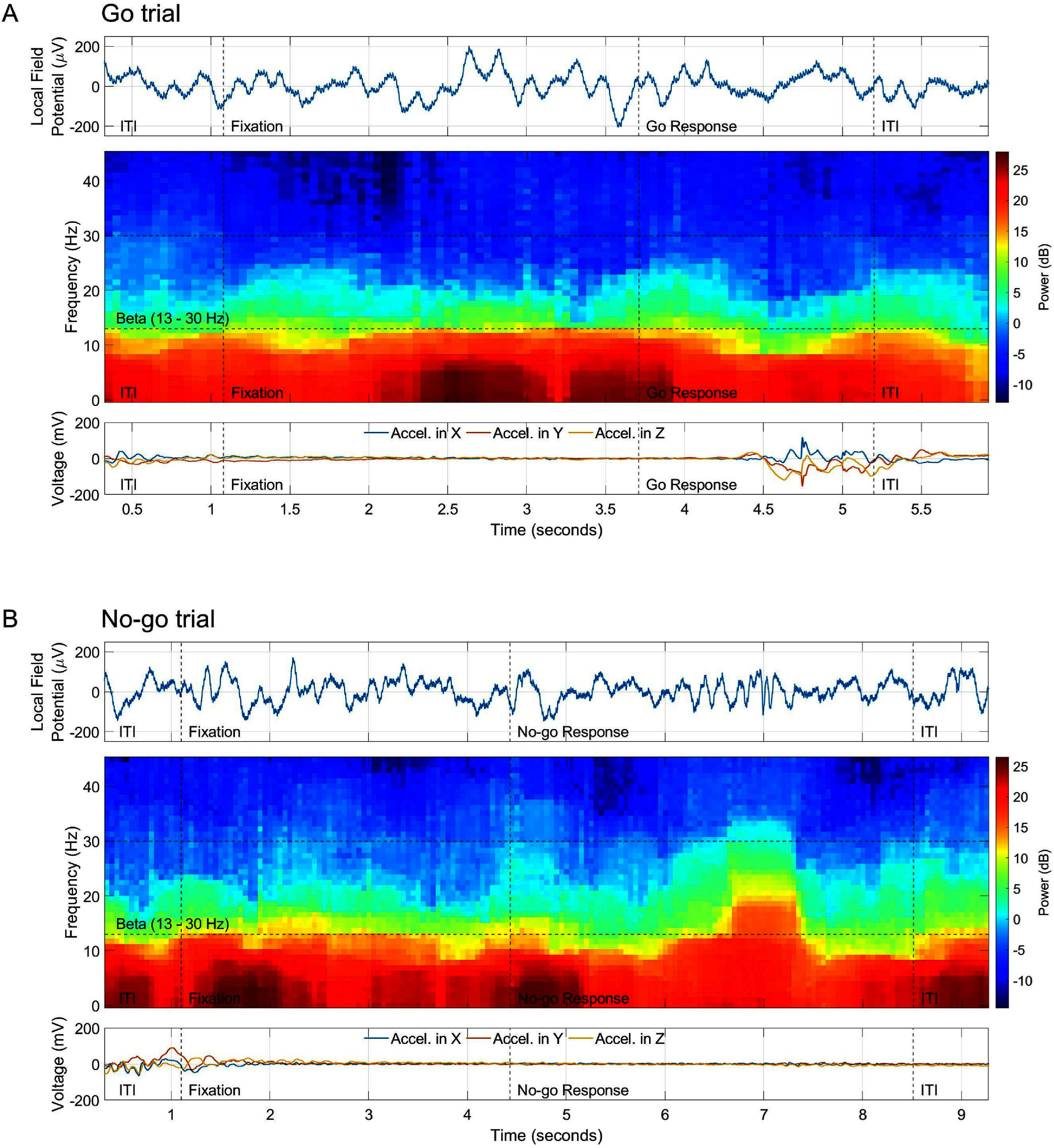
Comparison of (A) ‘Go’ and (B) ‘No-go’ trials across a consecutive inter-trial interval (ITI) on the same electrode contact in the right amygdala for participant ID 7. In both (A) and (B) panels, the first row depicts the LFP fluctuations over time, the middle row displays the spectrogram for the same period, and the third row shows voltage changes across the three axes of the accelerometer worn on the participant’s performing hand (right), all aligned with the ITI, fixation, and response phases of that trial. Differences in the temporal and spectral domain correspond with the execution of movement in the ‘Go’ trial (decrease of beta power) and withholding of movement in the ‘No-go’ trial (increase of beta power).

Signal quality was assessed under the guidance of an epileptologist. Detailed manual inspections were conducted to identify and exclude trials contaminated with interictal spikes. Tables 3 and 4 present the proportion of successful trials for both ‘Go’ and ‘No-go’ conditions after conducting signal quality assessment. Figure 2 features the LFPs and spectrograms of two illustrative examples from ‘Go’ and ‘No-go’ trials used in the analysis, contrasting the temporal and spectral differences between the two response conditions. All electrode contacts located in the left and right amygdala gray matter, identified from merged pre-operative MRI and post-operative CT scans, were included in the analysis.

**Table 3.** Summary of beta-band power modulation during the ‘Go’ response condition in the amygdala for all participants. The number of electrode contacts in the amygdala with significant modulation was determined under the Wilcoxon signed-rank test for multiple frequency bins within the beta-band, controlling for the false discovery rate. The percentage of contacts with significant modulation was based on the total number of number of contacts in the amygdala for that participant, as outlined in table 2. In the last column, the highest p-values are reported for participants who had contacts with a significant effect.

Electrode Contacts with significant modulation: Go response					
ID	Proportion of Successful Trials	Laterality	Beta-band (13–30 Hz)		
			Number and Percentage of contacts with Significant Modulation	Contact-Averaged Frequency Range of Significant Modulation (Hz)^a^	*p*-values
1	31/32	Contralateral	4 (100.0%)	13.2–28.3	*p* ⩽ 0.0455
	(96.9%)	Ipsilateral	4 (100.0%)	13.2–28.3	*p* ⩽ 0.0497

2	32/32	Contralateral	0 (0.0%)	—	—
	(100.0%)	Ipsilateral	2 (50.0%)	16.6–21.0	*p* ⩽ 0.0306

3	31/32	Contralateral	2 (50.0%)	27.8–28.8	*p* ⩽ 0.0387
	(96.9%)	Ipsilateral	0 (0.0%)	—	—

4	31/32	Contralateral	0 (0.0%)	—	—
	(96.9%)	Ipsilateral	2 (66.7%)	16.1–18.4	*p* ⩽ 0.0499

5	29/32	Contralateral	3 (100.0%)	27.8–28.8	*p* ⩽ 0.0024
	(90.6%)	Ipsilateral	3 (100.0%)	13.8–14.8	*p* ⩽ 0.0345

6	31/32	Ipsilateral	2 (50.0%)	22.9–29.8	*p* ⩽ 0.0167
	(96.9%)				

7	32/32	Ipsilateral	2 (50.0%)	13.2–17.6	*p* ⩽ 0.0402
	(100.0%)				

8	32/32	Contralateral	0 (0.0%)	—	—
	(100.0%)				

9	31/32	Contralateral	1 (25.0%)	16.0–17.0	*p* ⩽ 0.0021
	(96.9%)				

10	31/32	Contralateral	0 (0.0%)	—	—
	(96.9%)	Ipsilateral	3 (100.0%)	13.2–25.2	*p* ⩽ 0.0337

AVG	31.1/32	Contralateral	1.3 (34.4%)^b^	21.2–25.7^c^	—
	(97.2%)	Ipsilateral	2.3 (64.6%)^b^	15.6–22.2^c^	—

^a^ The contact-averaged frequency range of significant modulation is shown for the frequency range with the largest test-statistic sum in absolute value.

^b^ Average among all participants enrolled.

^c^ Average among participants with significant modulation.

**Table 4.** Summary of beta-band power modulation during the ‘No-go’ response condition in the amygdala for all participants. The number of electrode contacts in the amygdala with significant modulation was determined under the Wilcoxon signed-rank test for multiple frequency bins within the beta-band, controlling for the false discovery rate. The percentage of contacts with significant modulation was based on the total number of number of contacts in the amygdala for that participant, as outlined in table 2. In the last column, the highest *p*-values are reported for participants who had contacts with a significant effect.

Electrode contacts with significant modulation: No-go response					
ID	Proportion of successful trials	Laterality	Beta-band (13–30 Hz)		
			Number and Percentage of contacts with significant modulation	Contact-averaged frequency range of significant modulation (Hz)^a^	*p*-values
1	32/32	Contralateral	3 (75.0%)	14.5–26.9	*p* ⩽ 0.0349
	(100.0%)	Ipsilateral	4 (100.0%)	13.2–27.1	*p* ⩽ 0.034

2	32/32	Contralateral	4 (100.0%)	13.2–17.1	*p* ⩽ 0.0015
	(100.0%)	Ipsilateral	4 (100.0%)	13.2–17.1	*p* ⩽ 0.0324

3	32/32	Contralateral	0 (0.0%)	—	—
	(100.0%)	Ipsilateral	1 (33.3%)	16.1–17.1	*p* ⩽ 0.0124

4	32/32	Contralateral	0 (0.0%)	—	—
	(100.0%)	Ipsilateral	3 (100.0%)	16.4–17.7	*p* ⩽ 0.0176

5	32/32	Contralateral	2 (66.7%)	28.8–29.8	*p* ⩽ 0.0005
	(100.0%)	Ipsilateral	3 (100.0%)	16.4–20.3	*p* ⩽ 0.0494

6	27/32	Ipsilateral	1 (25.0%)	20.0–27.8	*p* ⩽ 0.0494
	(84.4%)				

7	25/32	Ipsilateral	1 (25.0%)	13.2–14.2	*p* ⩽ 0.0273
	(78.1%)				

8	17/32	Contralateral	0 (0.0%)	—	—
	(53.1%)				

9	25/32	Contralateral	4 (100.0%)	15.8–19.2	*p* ⩽ 0.0050
	(78.1%)				

10	24/32	Contralateral	0 (0.0%)	—	—
	(75.0%)	Ipsilateral	0 (0.0%)	—	—

AVG	27.8/32	Contralateral	1.6 (42.7%)^b^	18.1–23.3^c^	—
	(86.9%)	Ipsilateral	2.1 (60.4%)^b^	15.5–20.2^c^	—

^a^ The contact-averaged frequency range of significant modulation is shown for the frequency range with the largest test-statistic sum in absolute value.

^b^ Average among all participants enrolled.

^c^ Average among participants with significant modulation.

### 2.5. Spectral power analysis

We conducted multitaper spectral analysis, selected for its anti-leakage properties, for all trials of the experimental task with nine leading tapers and a time-bandwidth product of five. We used Chronux^©^ (version 2.12 v03, Woods Hole, MA, USA) for the spectral analysis with MATLAB^©^ 2021a. The multitaper method is an advanced spectral analysis technique that builds upon the classical fast Fourier transform (FFT) by providing a more accurate and reliable estimation of power spectra than traditional FFT methods that use a single window. This method applies multiple orthogonal tapers (window functions) to the same data set to reduce variance and spectral leakage more effectively than the single taper (window) approach used in a classical FFT analysis. Each tapered version of the data is then transformed using the FFT, and the resulting spectra are averaged to produce the final spectral estimate [41]. By leveraging multiple overlapping FFTs, each modified by a distinct window function, the multitaper method offers a sophisticated approach for analyzing complex signals, making it particularly suited for tasks requiring precise frequency-domain analysis.

To examine variations of beta-band power between the phases of the task, we calculated trial-averaged power spectral densities with bootstrapped (10 000 iterations) 95% confidence intervals for each phase of the experimental task. Each window of data used to compute trial-averaged spectral power was trimmed by a variable amount (between 0.1 and 1.75 s) at the beginning and end to exclude the phase transitions and maintain a consistent window size of 0.5 s for analysis. For the ITI, Fixation, and ‘No-go’ response phases, the analysis window was centered in the middle of the phase. For the ‘Go’ response phase, the window was aligned to the end of the response phase to capture the more stereotyped movement around the end of the reach to target. Confidence intervals were not used to determine statistically significant differences in beta-band power between phases. For statistical inference, a hypothesis test, described in the next section, was designed to assess significant differences and account for multiple comparisons between the frequency bins of the beta-band.

### 2.6. Statistical test of spectral power

Assessment of statistically significant differences in spectral power between phases (considering a significance level *α* = 0.05) was performed using the Wilcoxon-signed rank test for each frequency bin within the beta-band (13–30 Hz). The Wilcoxon signed-rank hypothesis test is a non-parametric test used for paired data that was selected because of the interdependency of the sample groups (ITI, fixation, and response) as they are obtained within the same trial. To assess significant beta-band power differences between the fixation (baseline) and response phases, we tested the null hypothesis that the difference in power spectral density between these two phases (PSD_Fix._—PSD_Res._) comes from a distribution with a median value of zero. The alternative hypothesis for the ‘Go’ response trials, that the data in (PSD_Fix._—PSD_Go Resp._) comes from a distribution with a median greater than zero (right-sided test), was used to assess significant decreases of beta-band power during the ‘Go’ condition. The alternative hypothesis for the ‘No-go’ response trials, that the data in (PSD_Fix._—PSD_No-go Resp._) comes from a distribution with median less than zero (left-sided test), was used to assess significant increases of beta-band power during the ‘No-go’ condition. To address the multiple comparisons problem, we conducted the Benjamini–Yekutieli (BY) procedure for controlling the false discovery rate, which controls under any dependency structure, in order to calculate the BY-corrected *p*-values [42].

### 2.7. Statistical test between amygdala regions contralateral and ipsilateral to the right (performing) hand

Statistical testing between amygdala regions contralateral and ipsilateral to the performing hand (left vs right hemispheres, respectively) was conducted to determine whether significant modulation in the beta-band power during the response phase was more prominent in one hemisphere. To account for differences in contact number and location among participants, a two-sided Yates-corrected *z*-test was used to evaluate the proportion of contacts with significant modulation in either the contralateral or ipsilateral sides of the amygdala. The proportions were normalized based on the total number of gray matter amygdala contacts implanted among all participants. In this analysis, the proportion of contacts with significant modulation was used to allow for comparisons between participants with varying numbers of total implanted contacts.

### 2.8. Statistical test between male and female sex

Statistical testing between males and females was conducted to determine if there was a significant difference in beta-band modulation in the amygdala related to sex. We used a two-sided Yates-corrected *z*-test to determine what proportion of contacts in the amygdala experienced beta-modulation in males and females for the ‘Go’ and ‘No-go’ tasks separately. Each proportion was normalized by the number of gray matter contacts in the amygdala implanted among all participants. The proportion of contacts showing significant modulation were used for comparisons between males and females.

## 3. Results

### 3.1. ‘Go’ response

**Figure 3.**
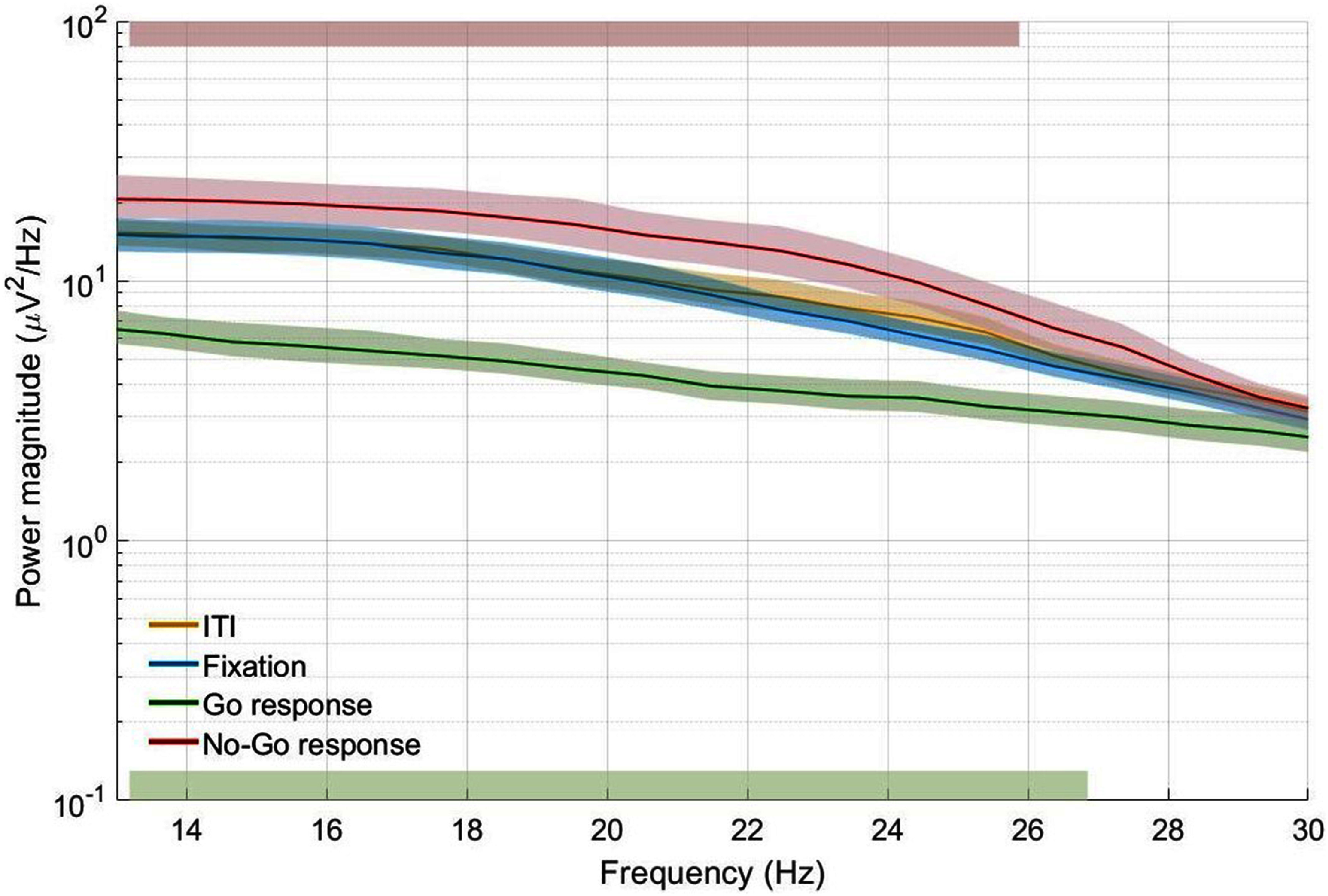
Trial-averaged power spectral density from an electrode contact in the amygdala in a representative participant (Solid lines) with bootstrapped 95% confidence intervals. Top red area: frequency interval with a significant increase of power during the ‘No-go’ response. Bottom green area: frequency interval with a significant decrease of power during the ‘Go’ response. ITI represents the inter-trial interval phase, during which there was no visual cue presented on the screen for 1–2 s.

In the ‘Go’ condition of the Go/No-go task, 9 out of 10 participants showed significant beta-band power decrease during the response phase in at least one electrode contact implanted in the amygdala in either hemisphere (*p* ⩽ 0.0499, Wilcoxon signed-rank test) when compared to the Fixation phase. Within this subset of nine participants, an average of 34.4% of the implanted contacts (1.3 contacts on average) in the contralateral amygdala showed significant beta-band power modulation, averaging between 21.2–25.7 Hz across all participants. In the ipsilateral side, an average of 64.6% of the implanted contacts (2.3 contacts on average) showed significant beta-band power modulation, averaging between 15.6–22.2 Hz across all participants. Figure 3 shows the trial-averaged power spectral densities for the averaged beta-band power modulation in the amygdala during the Go/No-go task for a representative participant. A summary of the observed beta-band power modulation in the ‘Go’ condition in both cerebral hemispheres of the amygdala for all participants is presented in table 3.

### 3.2. ‘No-go’ response

During the ‘No-go’ condition, 8 out of 10 participants showed significant beta-band power increase during the Response phase (*p* ⩽ 0.0494, Wilcoxon signed-rank test) when compared to the Fixation phase in at least one electrode contact implanted in the amygdala. In these eight participants, an average of 42.7% of the contacts in the contralateral side of the amygdala (1.6 contacts on average) showed significant beta-band power modulation, averaging between 18.1–23.3 Hz across all participants. In the ipsilateral amygdala, an average of 60.4% of the contacts (2.1 contacts on average) showed significant beta-band power modulation averaging between 15.5–20.2 Hz across all participants. A detailed presentation of the observed beta-band power modulation in the ‘No-go’ condition in both cerebral hemispheres of the amygdala for all participants can be found in table 4.

### 3.3. Contralateral vs ipsilateral amygdala (individual-level)

During the ‘Go’ condition, for 4 of 8 participants with electrode leads in the contralateral amygdala and 7 of 8 participants with leads in the ipsilateral side, power spectral analysis revealed a statistically significant decrease in power within contiguous regions of the beta-band. In the ‘No-go’ condition, 4 of 8 participants with electrode leads in the contralateral side and 7 of 8 participants with leads in the ipsilateral side presented a statistically significant increase in power of the beta-band during the ‘No-go’ Response phase compared with the fixation phase. In the contralateral hemisphere, 3 out of the 4 above mentioned participants showed beta-band modulation during both ‘Go’ and ‘No-go’ trials (participant IDs 1, 5, and 9). Of the 7 participants showing beta-band modulation in the ipsilateral side in either the ‘Go’ or ‘No-go’ tasks, 6 showed modulation during both (participant IDs 1, 2, 4, 5, 6, and 7).

### 3.4. Contralateral vs ipsilateral side of the amygdala (group-level, test of proportions)

Comparisons between the contralateral and ipsilateral side at group level were conducted for both ‘Go’ and ‘No-go’ conditions among all participants using the Yates-corrected *z*-test for proportions. In the ‘Go’ condition, 10 out of 28 (35.7%) contacts in the contralateral side from all participants showed a significant beta-band power decrease, while 18 out of 28 (64.3%) contacts in the ipsilateral side from all participants showed a significant beta-band power decrease. In the ‘No-go’ condition, 13 out of 28 (46.4%) contacts in the contralateral side from all participants showed a significant beta-band power increase, while 17 out of 28 (60.7%) contacts in the ipsilateral side showed a significant beta-band power increase. Although a higher percentage of contacts in the ipsilateral side presented significant beta-band power modulation than in the contralateral side in both the ‘Go’ and ‘No-go’ conditions, no significant differences were found in the proportion of contacts between contralateral vs ipsilateral sides using the Yates-corrected *z*-test (‘Go’ condition: *p* = 0.0614; ‘No-go’ condition: *p* = 0.4215).

### 3.5. Males vs. Females

In the ‘Go’ condition, 14 out of 30 electrodes (46.7%) implanted in female participants and 11 out of 26 electrodes (42.3%) implanted in male participants showed significant decreases in beta-band power between the fixation and response phases as determined by power spectral analysis. In the ‘No-go’ condition, 19 out of 30 electrodes (63.3%) implanted in female participants and 11 out of 26 electrodes (42.3%) implanted in male participants showed significant increases in beta-band modulation between fixation and response phases. No significant difference was found in the proportion of contacts experiencing beta modulation between males and females during both the ‘Go’ and ‘No-go’ tasks using the Yates-corrected *z*-test (‘Go’ condition: *p* = 0.954; ‘No-go’ condition: *p* = 0.192)

## 4. Discussion

Over the past few years, a growing body of evidence has suggested that the amygdala is involved in volitional motor control. In this study, we characterized beta-band power, known for its role in motor inhibition, in the amygdala during voluntary movements using a Go/No-go paradigm.

### 4.1. Amygdala during motor execution and inhibition

In this study, we observed that 90% of participants displayed significant beta-band power modulation in the amygdala during movement execution and 80% showed significant modulation when movement was withheld. Specifically, movement execution during the ‘Go’ task was associated with a decrease of beta-band power relative to the baseline period (Fixation phase), while movement inhibition during the ‘No-go’ task was associated with a relative increase of beta-band power. These observed effects are consistent with previous findings about the inhibitory role of the beta-band in other structures of the brain such as the pre-SMA [43], sensorimotor cortex [44], and STN [38, 45]. These findings are also in accordance with our previous studies regarding beta-band power modulation in the hippocampus during execution and inhibition of ARMs [22, 27].

To the best of our knowledge, no prior studies have directly characterized the role of the beta-band power in the amygdala during movement. Nevertheless, our findings are consistent with existing literature about the connections between the amygdala and motor cortex. Several studies using fMRI [46] and tractography [47] have shown the presence of structural connections between the amygdala and pre-SMA, which plays a role in movement inhibition. Similarly, a study conducted by Hassa *et al* in participants with conversion disorder discovered functional connections between the amygdala and pre-SMA [48]. Therefore, the effects of the beta-band may represent either direct modulation of motor activity by the amygdala or signal propagation from connected motor areas. Another study used a modified stop-signal task to show that increased activity in the amygdala due to fearful emotional stimuli resulted in prolonged reactive motor responses [10]. The same study also showed that amygdala activation was associated with activation of cortical structures not traditionally known to play roles in motor inhibition, suggesting that the amygdala may participate in circuit plasticity by modulating ongoing motor activity and recruiting other parts of the brain [10]. The presence of structural and functional connections between the amygdala and motor-related brain areas, such as the pre-SMA, suggest a potential pathway through which the amygdala could influence motor control. While our study focused on examining beta-band modulation during motor execution and inhibition, other studies such as those conducted by Tzagarakis *et al* have observed beta-band desynchronizations that may occur even prior to movement [49]. Therefore, these connections, coupled with the amygdala’s role in circuit plasticity and its ability to modulate motor activity in response to emotional stimuli, support the idea of some neural circuits that may become active prior to muscle activation, potentially contributing to the observed reduction in beta-band power. These circuits, involving the amygdala, may not only prepare the motor system for execution by reducing beta-band power but also adapt motor responses based on emotional and cognitive factors.

While most participants presented significant modulation in most of the electrode contacts implanted in the amygdala, there were exceptions and variability in the number of contacts with significant neural modulation. For example, participant 8 did not present beta-band modulation in any condition of the Go/No-go task. Additionally, participant 10 showed modulation only during the ‘Go’ task but not the ‘No-go’ task, and participant 3, 6, and 7 exhibited significant modulation in the ‘No-go’ condition but in a reduced number of contacts. This variability might stem from factors not controlled in this study, owing to clinical constraints that potentially influence neural modulation. For example, Ahmadi *et al* and Slinger *et al* found that the magnitude and evolution of beta-band power is highly affected by the progression of epilepsy in the human brain [50, 51], which may explain the underlying heterogeneity in our results. In addition, beta-band activity in epileptic brains has been shown to be modulated by medication levels [52], disease progression [53], and heterogeneity in brain function [54], all of which could not be controlled for in our study.

### 4.2. Amygdala laterality

In both ‘Go’ and ‘No-go’ conditions, more participants presented significant beta-band modulation in the ipsilateral side (7 out of 8 participants) versus the contralateral side (4 out of 8 participants). At a group level, we also observed a higher percentage of contacts in the ipsilateral side that showed significant modulation in both the ‘Go’ and ‘No-go’ conditions compared to the contralateral side. This effect is less likely due to handedness, as all but one participant (Participant 5) was right-handed, and Participant 5 displayed beta-band involvement in all electrodes for both the ‘Go’ and ‘No-go’ task. However, our data does not show enough evidence that a laterality effect in the amygdala is statistically significant, according to the results of the Yates-corrected *z*-test for proportions between contacts implanted ipsilateral versus contralateral to the performing (right) hand.

While we would expect right-hand movements to activate left-sided brain structures involved in motor control, there is some evidence that unilateral movement activates bilateral brain structures as well as regions outside the primary motor cortex [55, 56]. However, the amygdala was not mentioned in these studies due to its traditional role outside the motor circuit. Still, there is no clear consensus on the significance of amygdala lateralization. Recent theories, however, have focused on laterality as a result of function and response speed. For example, one meta-analysis showed that, across the studies they surveyed, the left amygdala was more active than the right during emotional processing regardless of stimulus type or valence [57]. Various studies have also posited that the left and right amygdala serve different functions in emotional processing. For example, Gläscher and Adolf hypothesized that while the right amygdala is more involved in rapid assessment of emotional stimuli, the left amygdala is more involved with higher-level processing and synthesis [58]. Similarly, Mathiak *et al* conducted a meta-analysis and determined that the right amygdala was more involved in the processing of facial images [59]. To our knowledge, this is the first study investigating the role of the amygdala in movement without providing a preceding emotional stimulus. However, our data do not show evidence of statistically significant laterality in the amygdala, hence not supporting the hypothesis regarding laterality in the amygdala during Go/No-go arm-reaching responses.

### 4.3. Gender and beta modulation

In this study, we did not observe a significant difference between males and females in the proportion of electrode contacts showing significant beta modulation in both the ‘Go’ and ‘No-go’ tasks. This finding is in contrast with our previous study about beta-modulation in the hippocampus, where we found a significantly higher proportion of electrode contacts that exhibited decreases in beta-band power during the ‘No-go’ task in females compared to males [27]. Sexual dimorphism in the amygdala has been established by various studies that have shown differences in functional connectivity between the amygdala and surrounding brain structures [60–62]. While we observed no sex-specific differences in amygdaloid beta modulation, recognizing how the amygdala contributes to motor control in males versus females is an area of further investigation. Future understanding of this will provide a better understanding of the role of the amygdala in motor execution and suppression.

### 4.4. Beta modulation in muscle coordination

Our findings provide further evidence that beta-band modulation plays a significant role in motor control [63] and are consistent with previous hypotheses that beta-band could reflect a motor state-change signal [64]. Furthermore, it has been shown that beta-band oscillation contributes to muscle [65], motor neuron unit firings [66], and cortico-muscular coherence [67]. However, its functional role in muscle coordination remains unclear. Kilner *et al* examined the oscillatory coherence between the simultaneously recorded magnetoencephalogram in motor cortex and electromyogram in hand and forearm muscles during steady grip of a hold task [5]. They found that maximum coherence within the beta-band frequency range corresponded to subjects maintaining a steady grip, with a subsequent disappearance of coherence during movement. De Lange *et al* observed a similar phenomenon during the execution of imagined movements [68]. Reyes *et al* expanded upon these studies and found that beta-band cortico-muscular coherence with hand muscles was significantly reduced when greater individuated control was required during a spring compression motor task [67]. Thus, beta-band frequency oscillations may not be essential for driving the force behind task performance [69] but rather they may reflect a form of fine-tuned motor control. Ultimately, the role of cortical and subcortical beta-band oscillatory activity in muscle function remains an area requiring future investigation.

### 4.5. Limitations

Our study has certain limitations to consider. Firstly, our participant pool consisted of individuals with chronic epilepsy, a condition known to affect neurophysiological signaling, which may have affected the generalizability of our results. In addition, abnormalities of the amygdala have been described in participants with epilepsy, including *in vitro* spontaneous electrical discharges [70], neurotransmitter redistribution [71], and amygdaloid volume loss [72]. Therefore, it is unknown if the beta-band modulation observed in this study would also be seen in non-epileptic participants. Thirdly, electrodes were placed in the brain according to clinical circumstances, and the resultant heterogeneity in signal location may have affected interpretation of our findings. However, we attempted to control for this possibility by conducting several trials and averaging our results. In terms of potential confounders, while it was not possible to have full control over eye movement, we implemented measures to mitigate its impact. For example, participants were explicitly instructed to fix their gaze on the dots displayed on the screen, potentially reducing eye movements. Notably, incidental eye movements were most likely to occur during phase transitions (where the fixation dot and target appear or disappear), which were excluded from the analysis.

Additionally, even though the reference white matter contact was subtracted from all channels to reduce common noise, it is still possible that part of the observed beta-band modulation may have been conducted from nearby structures that participate in motor control, such as the motor cortex [73], plausibly through volume conduction [74]. This is particularly more probable for electrode contacts farther from the reference contact, where the signals have less temporal similarity. However, we aimed to carefully choose a reference contact in white matter that would provide high signal quality based on our own visual inspection of the data and an epileptologist’s assessment.

### 4.6. Future directions

Through this study, we observed significant modulation in the beta-band in the amygdala during a Go/No-go voluntary movement task. The finding of beta-induced motor inhibition is consistent with previous studies that found similar behavior in other parts of the brain. The additional finding of laterality in the amygdala during these movement tasks, although not statistically significant, is in line with known differences between the right and left amygdala during emotional processing. However, the significance of this finding is yet unknown and may be a topic of future investigation. We believe this study is the first to investigate beta-band power from direct electrophysiology of the amygdala during a direct movement task. Future research will further examine how amygdala beta-band oscillations modulate during initiation, execution, and suppression of voluntary motor activity, as well as investigate neural tuning based on target direction, which would require increasing the number of trials per target direction.

While the scope of this study was specifically focused on the overall beta band (13–30 Hz) to systematically investigate its broad role in motor control as an initial step, examining modulation differences within other frequency bands, such as the alpha and gamma bands, and distinctions within the low- and high-beta bands [75], could yield a more comprehensive understanding of the spectral dynamics in motor control, building upon the findings of the present study and previous studies in gamma band modulation in the amygdala during similar tasks [36]. These future directions could not only help in delineating the specific contributions of different frequency bands to motor control but also deepen our understanding of the complex neural mechanisms involved in motor execution and inhibition.

## 5. Conclusions

Our work reports the novel finding of beta-band power modulation in the human amygdala during voluntary movement in the setting of motor execution versus inhibition. This finding adds to previous studies in other brain areas that have linked beta-band power to motor control. The presence of beta-band power modulation in these conditions suggests that the beta-band power from electrode contacts in the amygdala can potentially differentiate between motor inhibition and execution. Our study additionally highlights an overall tendency in laterality of beta-band power in the human amygdala (*R* > *L*), although this trend did not reach statistical significance based on the Yates-corrected *z*-test for proportions. This laterality effect is less likely to be due to handedness given all but one of our participant population was right-handed. Considering the subtle inclination towards laterality in the amygdala, this study presents an opportunity to delve deeper into the characterization of amygdaloid beta-band power laterality in the setting of motor processing.

## Data Availability

The data cannot be made publicly available upon publication because no suitable repository exists for hosting data in this field of study. The data that support the findings of this study are available upon reasonable request from the authors.
